# Treatment strategies for asthma: reshaping the concept of asthma management

**DOI:** 10.1186/s13223-020-00472-8

**Published:** 2020-08-15

**Authors:** Alberto Papi, Francesco Blasi, Giorgio Walter Canonica, Luca Morandi, Luca Richeldi, Andrea Rossi

**Affiliations:** 1 Section of Cardiorespiratory and Internal Medicine, Department of Morphology, Surgery and Experimental Medicine, University of Ferrara, Ferrara, Italy; 2 Internal Medicine Department, Respiratory Unit and Adult Cystic Fibrosis Center, Fondazione IRCCS Cà Granda Ospedale Maggiore Policlinico, Milan, Italy; 3 Department of Pathophysiology and Transplantation, Università degli Studi di Milano, Milan, Italy; 4 Personalized Medicine Asthma & Allergy Clinic, Humanitas University & Istituto Clinico Humanitas, Milan, Italy; 5 Università Cattolica del Sacro Cuore, Fondazione Policlinico A. Gemelli IRCCS, Rome, Italy; 6 Respiratory Section, Department of Medicine, University of Verona, Verona, Italy; 7 Respiratory Unit, Emergency Department, University Hospital S. Anna, Via Aldo Moro 8, 44124 Ferrara, Italy

**Keywords:** Asthma, Anti-inflammatory treatment, Disease control, Patient outcomes

## Abstract

Asthma is a common chronic disease characterized by episodic or persistent respiratory symptoms and airflow limitation. Asthma treatment is based on a stepwise and control-based approach that involves an iterative cycle of assessment, adjustment of the treatment and review of the response aimed to minimize symptom burden and risk of exacerbations. Anti-inflammatory treatment is the mainstay of asthma management. In this review we will discuss the rationale and barriers to the treatment of asthma that may result in poor outcomes. The benefits of currently available treatments and the possible strategies to overcome the barriers that limit the achievement of asthma control in real-life conditions and how these led to the GINA 2019 guidelines for asthma treatment and prevention will also be discussed.

## Background

Asthma, a major global health problem affecting as many as 235 million people worldwide [1], is a common, non-communicable, and variable chronic disease that can result in episodic or persistent respiratory symptoms (e.g. shortness of breath, wheezing, chest tightness, cough) and airflow limitation, the latter being due to bronchoconstriction, airway wall thickening, and increased mucus.

The pathophysiology of the disease is complex and heterogeneous, involving various host-environment interactions occurring at various scales, from genes to organ [2].

Asthma is a chronic disease requiring ongoing and comprehensive treatment aimed to reduce the symptom burden (i.e. good symptom control while maintaining normal activity levels), and minimize the risk of adverse events such as exacerbations, fixed airflow limitation and treatment side effects [3, 4].

Asthma treatment is based on a stepwise approach. The management of the patient is control-based; that is, it involves an iterative cycle of assessment (e.g. symptoms, risk factors, etc.), adjustment of treatment (i.e. pharmacological, non-pharmacological and treatment of modifiable risk factors) and review of the response (e.g. symptoms, side effects, exacerbations, etc.). Patients’ preferences should be taken into account and effective asthma management should be the result of a partnership between the health care provider and the person with asthma, particularly when considering that patients and clinicians might aim for different goals [4].

This review will discuss the rationale and barriers to the treatment of asthma, that may result in poor patient outcomes. The benefits of currently available treatments and the possible strategies to overcome the barriers that limit the achievement of asthma control in real-life situations will also be discussed.

### The treatment of asthma: where are we? Evolution of a concept

Asthma control medications reduce airway inflammation and help to prevent asthma symptoms; among these, inhaled corticosteroids (ICS) are the mainstay in the treatment of asthma, whereas quick-relief (reliever) or rescue medicines quickly ease symptoms that may arise acutely. Among these, short-acting beta-agonists (SABAs) rapidly reduce airway bronchoconstriction (causing relaxation of airway smooth muscles).

National and international guidelines have recommended SABAs as first-line treatment for patients with mild asthma, since the Global Initiative for Asthma guidelines (GINA) were first published in 1995, adopting an approach aimed to control the symptoms rather than the underlying condition; a SABA has been the recommended rescue medication for rapid symptom relief. This approach stems from the dated idea that asthma symptoms are related to bronchial smooth muscle contraction (bronchoconstriction) rather than a condition concomitantly caused by airway inflammation. In 2019, the GINA guidelines review (GINA 2019) [4] introduced substantial changes overcoming some of the limitations and “weaknesses” of the previously proposed stepwise approach to adjusting asthma treatment for individual patients. The concept of an anti-inflammatory reliever has been adopted at all degrees of severity as a crucial component in the management of the disease, increasing the efficacy of the treatment while lowering SABA risks associated with patients’ tendency to rely or over-rely on the as-needed medication.

Until 2017, the GINA strategy proposed a pharmacological approach based on a controller treatment (an anti-inflammatory, the pillar of asthma treatment), with a SABA as an additional rescue intervention. The reliever, a short-acting bronc hodilator, was merely an *addendum*, a medication to be used in case the real treatment (the controller) failed to maintain disease control: SABAs effectively induce rapid symptom relief but are ineffective on the underlying inflammatory process. Based on the requirement to achieve control, the intensity of the controller treatment was related to the severity of the disease, varying from low-dose ICS to combination low-dose ICS/long-acting beta-agonist (LABA), medium-dose ICS/LABA, up to high-dose ICS/LABA, as preferred controller choice, with a SABA as the rescue medication. As a result, milder patients were left without any anti-inflammatory treatment and could only rely on SABA rescue treatment.

Poor adherence to therapy is a major limitation of a treatment strategy based on the early introduction of the regular use of controller therapy [5]. Indeed, a number of surveys have highlighted a common pattern in the use of inhaled medication [6], in which treatment is administered only when asthma symptoms occur; in the absence of symptoms, treatment is avoided as patients perceive it as unnecessary. When symptoms worsen, patients prefer to use reliever therapies, which may result in the overuse of SABAs [7]. Indirect evidence suggests that the overuse of beta-agonists alone is associated with increased risk of death from asthma [8].

In patients with mild persistent disease, low-dose ICS decreases the risk of severe exacerbations leading to hospitalization and improves asthma control [9]. When low-dose ICS are ineffective in controlling the disease (Step 3 of the stepwise approach), a combination of low-dose ICS with LABA maintenance was the recommended first-choice treatment, plus as-needed SABA [3, 10]. Alternatively, the combination low-dose ICS/LABA (formoterol) was to be used as single maintenance and reliever treatment (SMART). The SMART strategy containing the rapid-acting formoterol was recommended throughout GINA Steps 3 to 5 based on solid clinical-data evidence [3].

The addition of a LABA to ICS treatment reduces both severe and mild asthma exacerbation rates, as shown in the one-year, randomized, double-blind, parallel-group FACET study [11]. This study focused on patients with persistent asthma symptoms despite receiving ICS and investigated the efficacy of the addition of formoterol to two dose levels of budesonide (100 and 400 µg *bid*) in decreasing the incidence of both severe and mild asthma exacerbations. Adding formoterol decreased the incidence of both severe and mild asthma exacerbations, independent of ICS dose. Severe and mild exacerbation rates were reduced by 26% and 40%, respectively, with the addition of formoterol to the lower dose of budesonide; the corresponding reductions were 63% and 62%, respectively, when formoterol was added to budesonide at the higher dose.

The efficacy of the ICS/LABA combination was confirmed in the post hoc analysis of the FACET study, in which patients were exposed to a combination of formoterol and low-dose budesonide [12]. However, such high levels of asthma control are not achieved in real life [5]. An explanation for this is that asthma is a variable condition and this variability might include the exposure of patients to factors which may cause a transient steroid insensitivity in the inflammatory process. This, in turn, may lead to an uncontrolled inflammatory response and to exacerbations, despite optimal controller treatment. A typical example of this mechanism is given by viral infections, the most frequent triggers of asthma exacerbations. Rhinoviruses, the most common viruses found in patients with asthma exacerbations, interfere with the mechanism of action of corticosteroids making the anti-inflammatory treatment transiently ineffective. A transient increase in the anti-inflammatory dose would overcome the trigger-induced anti-inflammatory resistance, avoiding uncontrolled inflammation leading to an exacerbation episode [13–15].

**Fig. 1:**
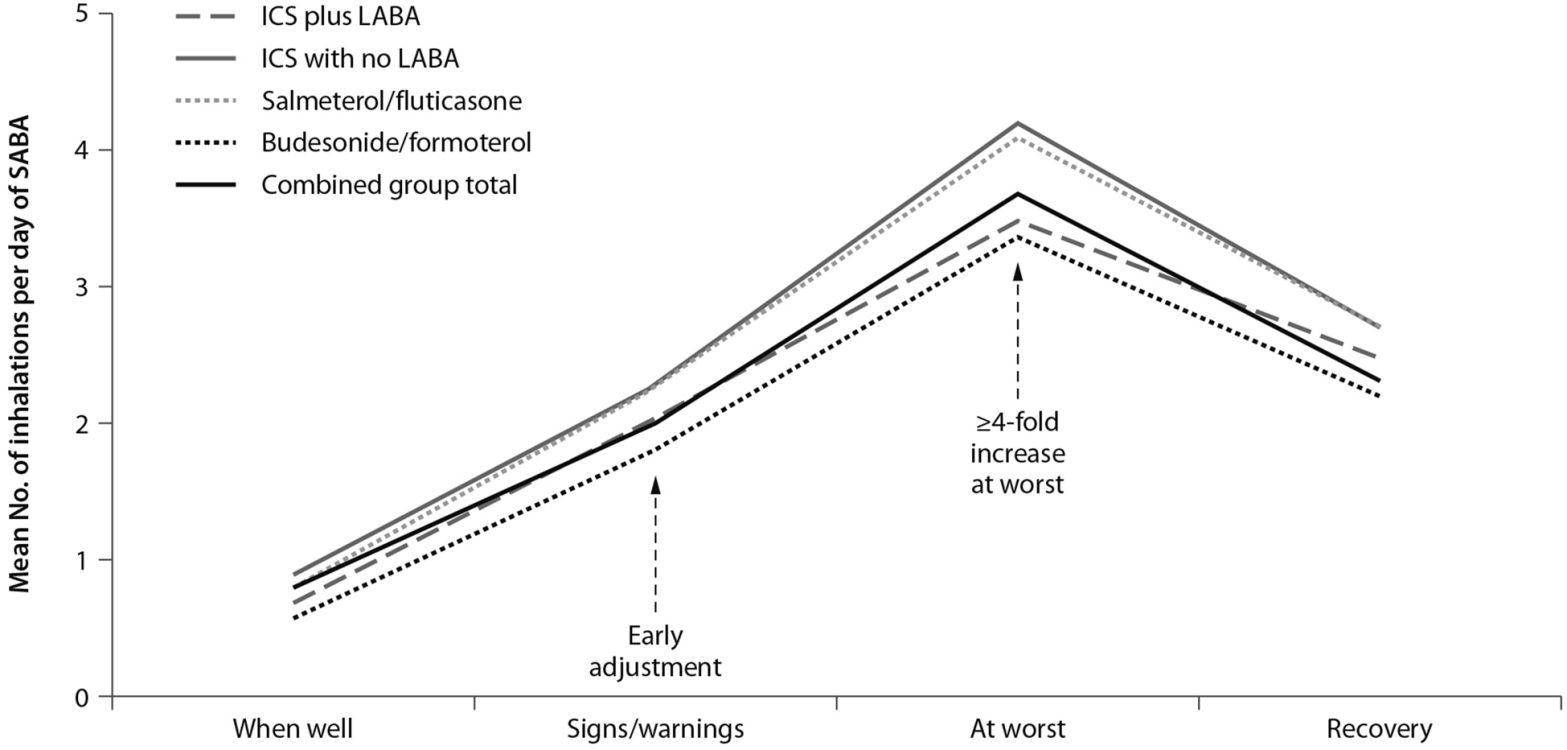
Mean use of SABA at different stages of asthma worsening. Patients have been grouped according to maintenance therapy shown in the legend. From [17], modified

Indeed, symptoms are associated with worsening inflammation and not only with bronchoconstriction. Romagnoli et al. showed that inflammation, as evidenced by sputum eosinophilia and eosinophilic markers, is associated with symptomatic asthma [16]. A transient escalation of the ICS dose would prevent loss of control over inflammation and decrease the risk of progression toward an acute episode. In real life, when experiencing a deterioration of asthma control, patients self-treat by substantially increasing their SABA medication (Fig. 1); it is only subsequently that they (modestly) increase the maintenance treatment [17].

As bronchodilators, SABAs do not control the underlying inflammation associated with increased symptoms. The “as required” use of SABAs is not the most effective therapeutic option in controlling a worsening of inflammation, as signaled by the occurrence of symptoms; instead, an anti-inflammatory therapy included in the rescue medication along with a rapid-acting bronchodilator could provide both rapid symptom relief and control over the underlying inflammation. Thus, there is a need for a paradigm shift, a new therapeutic approach based on the rescue use of an inhaled rapid-acting beta-agonist combined with an ICS: an anti-inflammatory reliever strategy [18].

**Fig. 2:**
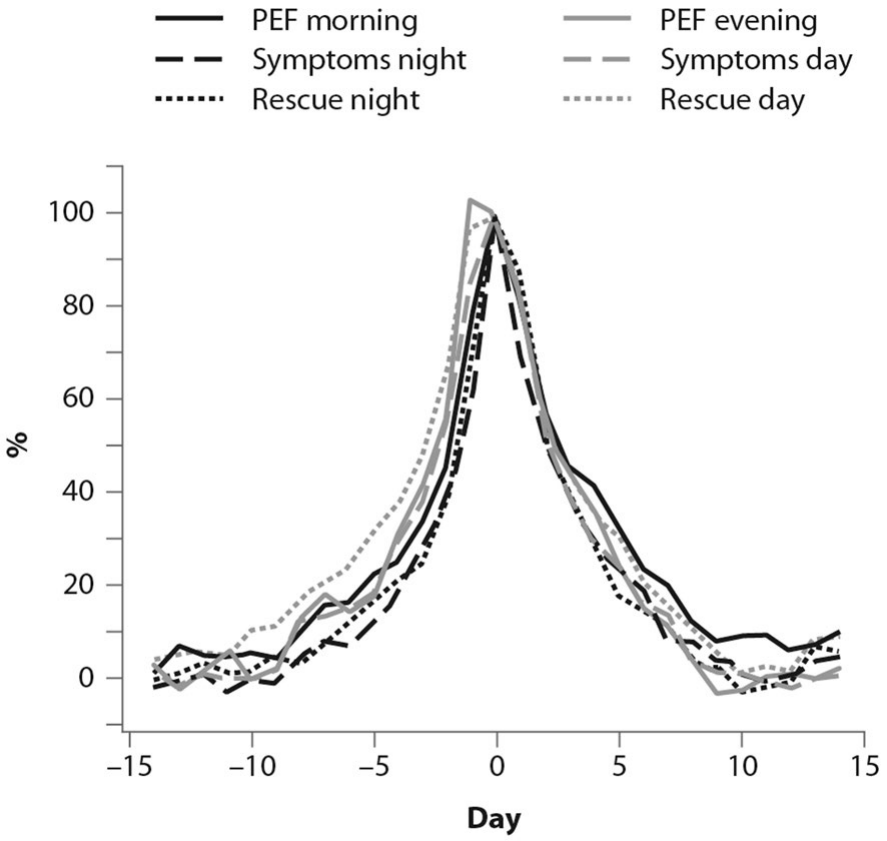
Percent variation in symptoms, rescue beta-agonist use and peak expiratory flow (PEF) during an exacerbation. In order to allow comparison over time, data have been standardized (Day-14 = 0%; maximum change = 100%) (From [19])

The symptoms of an exacerbation episode, as reported by Tattersfield and colleagues in their extension of the FACET study, increase gradually before the peak of the exacerbation (Fig. 2); and the best marker of worsening asthma is the increased use of rescue beta-agonist treatment that follows exactly the pattern of worsening symptomatology [19]. When an ICS is administered with the rescue bronchodilator, the patient would receive anti-inflammatory therapy when it is required; that is, when the inflammation is uncontrolled, thus increasing the efficiency of the anti-inflammatory treatment.

### Barriers and paradoxes of asthma management

A number of barriers and controversies in the pharmacological treatment of asthma have prevented the achievement of effective disease management [20]. O’Byrne and colleagues described several such controversies in a commentary published in 2017, including: (1) the recommendation in Step 1 of earlier guidelines for SABA bronchodilator use alone, despite asthma being a chronic inflammatory condition; and (2) the autonomy given to patients over perception of need and disease control at Step 1, as opposed to the recommendation of a fixed-dose approach with treatment-step increase, regardless of the level of symptoms [20]. Other controversies outlined were: (3) a difficulty for patients in understanding the recommendation to minimize SABA use at Step 2 and switch to a fixed-dose ICS regimen, when they perceive SABA use as more effective; (4) apparent conflicting safety messages within the guidelines that patient-administered SABA monotherapy is safe, but patient-administered LABA monotherapy is not; and (5) a discrepancy as to patients’ understanding of “controlled asthma” and their symptom frequency, impact and severity [20].

Controversies (1) and (2) can both establish an early over-dependence on SABAs. Indeed, asthma patients freely use (and possibly overuse) SABAs as rescue medication. UK registry data have recently suggested SABA overuse or overreliance may be linked to asthma-related deaths: among 165 patients on short-acting relievers at the time of death, 56%, 39%, and 4% had been prescribed > 6, > 12, and > 50 SABA inhalers respectively in the previous year [21]. Registry studies have shown the number of SABA canisters used per year to be directly related to the risk of death in patients with asthma. Conversely, the number of ICS canisters used per year is inversely related to the rate of death from asthma, when compared with non-users of ICS [8, 22]. Furthermore, low-dose ICS used regularly are associated with a decreased risk of asthma death, with discontinuation of these agents possibly detrimental [22].

Other barriers to asthma pharmacotherapy have included the suggestion that prolonged treatment with LABAs may mask airway inflammation or promote tolerance to their effects. Investigating this, Pauwels and colleagues found that in patients with asthma symptoms that were persistent despite taking inhaled glucocorticoids, the addition of regular treatment with formoterol to budesonide for a 12-month period did not decrease asthma control, and improved asthma symptoms and lung function [11].

### Treatment strategies across all levels of asthma severity

Focusing on risk reduction, the 2014 update of the GINA guidelines recommended as-needed SABA for Step 1 of the stepwise treatment approach, with low-dose ICS maintenance therapy as an alternative approach for long-term anti-inflammatory treatment [23]. Such a strategy was only supported by the evidence from a post hoc efficacy analysis of the START study in patients with recently diagnosed mild asthma [24]. The authors showed that low-dose budesonide reduced the decline of lung-function over 3 years and consistently reduced severe exacerbations, regardless of symptom frequency at baseline, even in subjects with symptoms below the then-threshold of eligibility for ICS [24]. However, as for all post hoc analyses, the study by Reddel and colleagues does not provide conclusive evidence and, even so, their results could have questionable clinical significance for the management of patients with early mild asthma. To be effective, this approach would require patients to be compliant to regular twice-daily ICS for 10 years to have the number of exacerbations reduce by one. In real life, it is highly unlikely that patients with mild asthma would adhere to such a regular regimen [25].

**Fig. 3:**
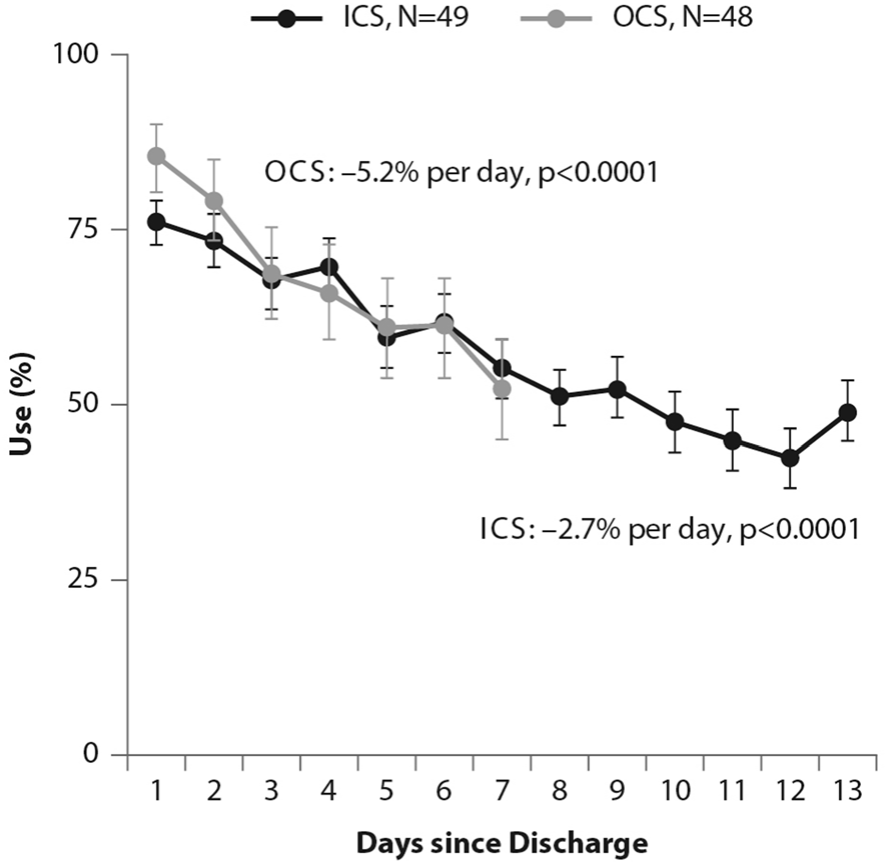
Use of inhaled (ICS) and oral (OCS) corticosteroids in patients after hospital discharge among high-risk adult patients with asthma. The corticosteroid use was monitored electronically. Error bars represent the standard errors of the measured ICS and OCS use (From [27])

The 2016 update to the GINA guidelines lowered the threshold for the use of low-dose ICS (GINA Step 2) to two episodes of asthma symptoms per month (in the absence of any supportive evidence for the previous cut-off). The objective was to effectively increase the asthma population eligible to receive regular ICS treatment and reduce the population treated with a SABA only, given the lack of robust evidence of the latter’s efficacy and safety and the fact that asthma is a variable condition characterized by acute exacerbations [26]. Similarly, UK authorities recommended low-dose ICS treatment in mild asthma, even for patients with suspected asthma, rather than treatment with a SABA alone [10]. However, these patients are unlikely to have good adherence to the regular use of an ICS. It is well known that poor adherence to treatment is a major problem in asthma management, even for patients with severe asthma. In their prospective study of 2004, Krishnan and colleagues evaluated the adherence to ICS and oral corticosteroids (OCS) in a cohort of patients hospitalized for asthma exacerbations [27]. The trend in the data showed that adherence to ICS and OCS treatment in patients dropped rapidly to reach nearly 50% within 7 days of hospital discharge, with the rate of OCS discontinuation per day nearly double the rate of ICS discontinuation per day (− 5.2% vs. − 2.7%; p < 0.0001 respectively, Fig. 3), thus showing that even after a severe event, patients’ adherence to treatment is suboptimal [27].

Guidelines set criteria with the aim of achieving optimal control of asthma; however, the attitude of patients towards asthma management is suboptimal. Partridge and colleagues were the first in 2006 to evaluate the level of asthma control and the attitude of patients towards asthma management. Patients self-managed their condition using their medication as and when they felt the need, and adjusted their treatment by increasing their intake of SABA, aiming for an immediate relief from symptoms [17]. The authors concluded that the adoption of a patient-centered approach in asthma management could be advantageous to improve asthma control.

The concomitant administration of an as-needed bronchodilator and ICS would provide rapid relief while administering anti-inflammatory therapy. This concept is not new: in the maintenance and reliever approach, patients are treated with ICS/formoterol (fast-acting, long-acting bronchodilator) combinations for both maintenance and reliever therapy. An effective example of this therapeutic approach is provided in the SMILE study in which symptomatic patients with moderate to severe asthma and treated with budesonide/formoterol as maintenance therapy were exposed to three different as-needed options: SABA (terbutaline), rapid-onset LABA (formoterol) and a combination of LABA and ICS (budesonide/formoterol) [28]. When compared with formoterol, budesonide/formoterol as reliever therapy significantly reduced the risk of severe exacerbations, indicating the efficacy of ICS as rescue medication and the importance of the as-needed use of the anti-inflammatory reliever.

**Fig. 4:**
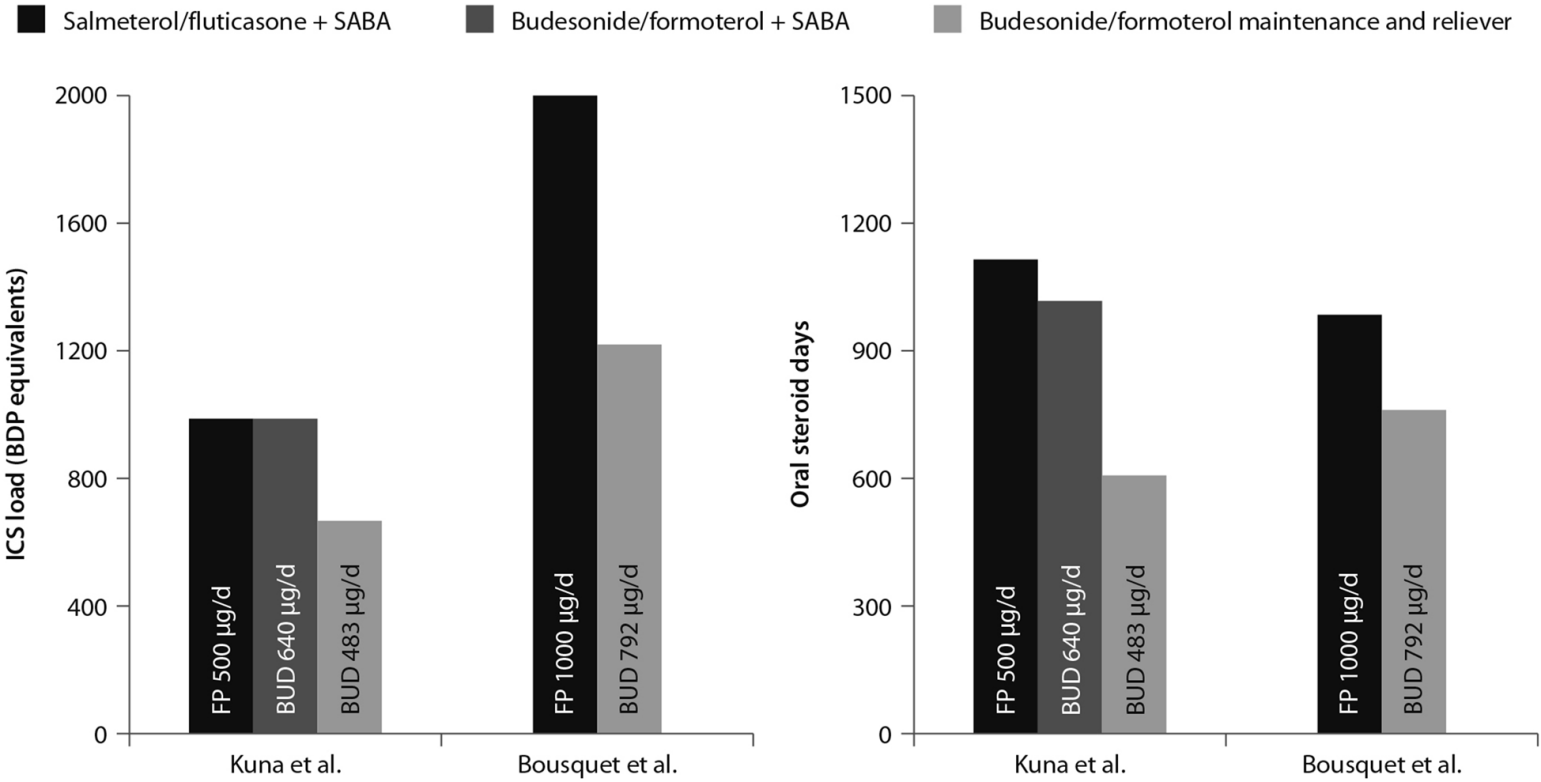
Comparison between the improvements in daily asthma control resulting from the use of budesonide/formoterol maintenance and reliever therapy vs. higher dose of ICS/LABA + SABAZ and steroid load for the two regimens (Data from [29, 30])

The combination of an ICS and a LABA (budesonide/formoterol) in one inhaler for both maintenance and reliever therapy is even more effective than higher doses of maintenance ICS and LABA, as evidenced by Kuna and colleagues and Bousquet and colleagues (Fig. 4) [29, 30].

The effects of single maintenance and reliever therapy versus ICS with or without LABA (controller therapy) and SABA (reliever therapy) have been recently addressed in the meta-analysis by Sobieraj and colleagues, who analysed 16 randomized clinical trials involving patients with persistent asthma [31]. The systematic review supported the use of single maintenance and reliever therapy, which reduces the risk of exacerbations requiring systemic corticosteroids and/or hospitalization when compared with various strategies using SABA as rescue medication [31].

This concept was applied to mild asthma by the BEST study group, who were the first to challenge the regular use of ICS. A pilot study by Papi and colleagues evaluated the efficacy of the symptom-driven use of beclomethasone dipropionate plus albuterol in a single inhaler versus maintenance with inhaled beclomethasone and as-needed albuterol. In this six-month, double-blind, double-dummy, randomized, parallel-group trial, 455 patients with mild asthma were randomized to one of four treatment groups: an as-needed combination therapy of placebo *bid* plus 250 μg of beclomethasone and 100 μg of albuterol in a single inhaler; an as-needed albuterol combination therapy consisting of placebo *bid* plus 100 μg of albuterol; regular beclomethasone therapy, comprising beclomethasone 250 μg *bid* and 100 μg albuterol as needed); and regular combination therapy with beclomethasone 250 μg and albuterol 100 μg in a single inhaler *bid* plus albuterol 100 μg as needed.

The rescue use of beclomethasone/albuterol in a single inhaler was as efficacious as the regular use of inhaled beclomethasone (250 μg *bid*) and it was associated with a lower 6-month cumulative dose of the ICS [32].

**Fig. 5:**
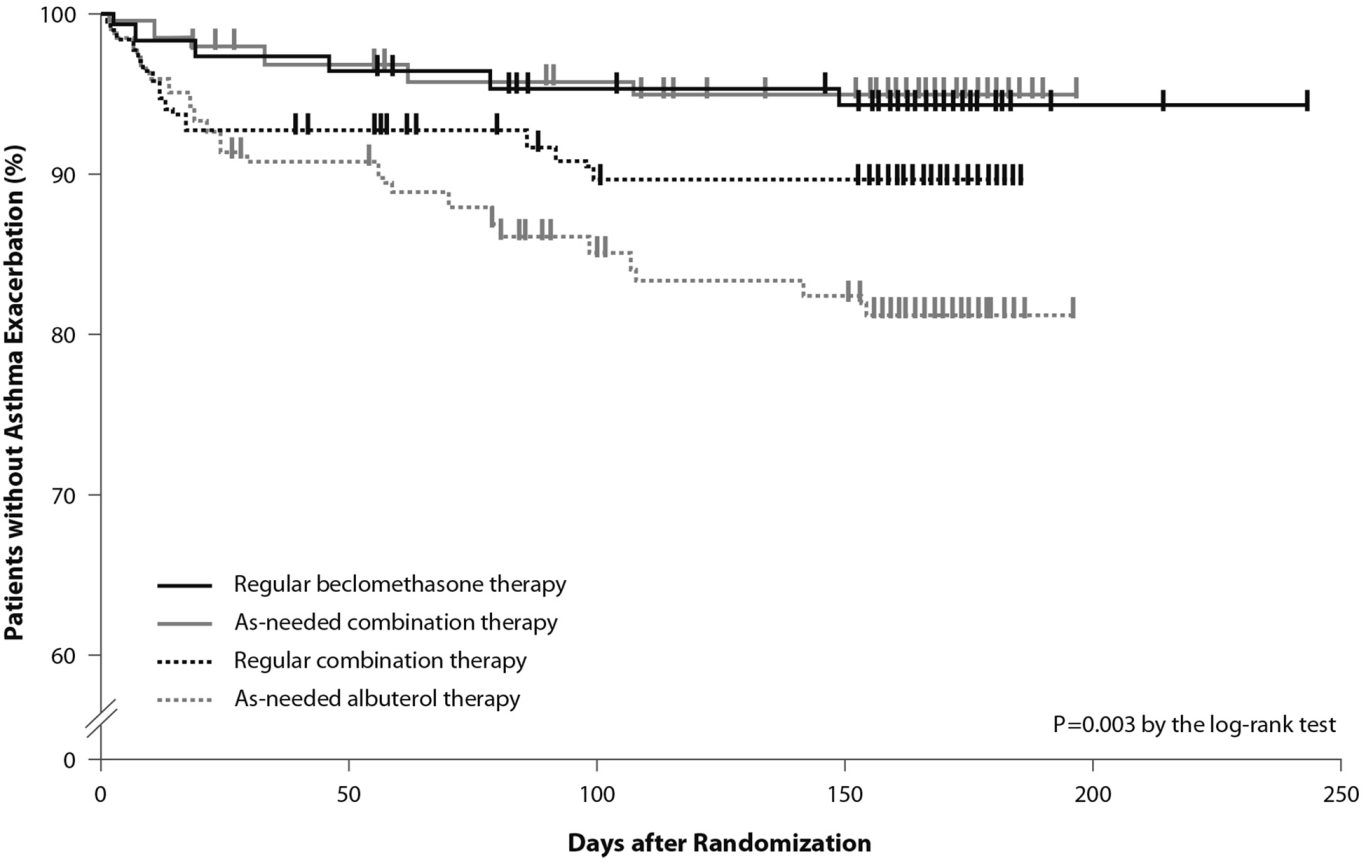
Kaplan Meier analysis of the time to first exacerbation (modified intention-to-treat population). First asthma exacerbations are shown as thick marks. As-needed albuterol therapy = placebo bid plus 100 μg of albuterol as needed; regular combination therapy = 250 μg of beclomethasone and 100 μg of albuterol in a single inhaler bid plus 100 μg of albuterol as needed; regular beclomethasone therapy = 250 μg of beclomethasone bid and 100 μg of albuterol as needed; as-needed combination therapy = placebo bid plus 250 μg of beclomethasone and 100 μg of albuterol in a single inhaler as needed (From [32])

The time to first exacerbation differed significantly among groups (*p *= 0.003), with the shortest in the as-needed albuterol and placebo group (Fig. 5). Figure 5 also shows equivalence between the as-needed combination therapy and the regular beclomethasone therapy. However, these results were not conclusive since the study was not powered to evaluate the effect of the treatment on exacerbations. In conclusion, as suggested by the study findings, mild asthma patients may require the use of an as-needed ICS and an inhaled bronchodilator rather than a regular treatment with ICS [32].

### Moving forward: a new approach to the management of asthma patients

Nearly a decade after the publication of the BEST study in 2007, the use of this alternative therapeutic strategy was addressed in the SYGMA 1 and SYGMA 2 trials. These double-blind, randomized, parallel-group, 52-week phase III trials evaluated the efficacy of as-needed use of combination formoterol (LABA) and the ICS budesonide as an anti-inflammatory reliever in patients requiring GINA Step 2 treatment, with the current reliever therapy (e.g. as-needed SABA) or with low-dose maintenance ICS (inhaled budesonide *bid*) plus as-needed SABA, administered as regular controller therapy [33, 34].

The SYGMA 1 trial, which enrolled 3849 patients, aimed to demonstrate the superiority of the as-needed use of the combination budesonide/formoterol over as-needed terbutaline, as measured by the electronically-recorded proportion of weeks with well-controlled asthma [34]. The more pragmatic SYGMA 2 trial enrolled 4215 patients with the aim to demonstrate that the budesonide/formoterol combination is non-inferior to budesonide plus as-needed terbutaline in reducing the relative rate of annual severe asthma exacerbations [33]. Both trials met their primary efficacy outcomes. In particular, as-needed budesonide/formoterol was superior to as-needed SABA in controlling asthma symptoms (34.4% versus 31.1%) and preventing exacerbations, achieving a 64% reduction in exacerbations. In both trials, budesonide/formoterol as-needed was similar to budesonide maintenance *bid* at preventing severe exacerbations, with a substantial reduction of the inhaled steroid load over the study period (83% in the SYGMA 1 trial and 75% in the SYGMA 2 trial). The time to first exacerbation did not differ significantly between the two regimens; however, budesonide/formoterol was superior to SABA in prolonging the time to first severe exacerbation [33, 34].

The double-blind, placebo-controlled design of the SYGMA trials does not fully address the advantages of anti-inflammatory reliever strategy in patients who often rely on SABAs for symptom relief, so to what extent the study findings could apply to real-life practice settings was unclear.

These limitations were overcome by the results of the Novel START study, an open-label, randomized, parallel-group, controlled trial designed to reflect real-world practice, which demonstrated the effectiveness in mild asthma of budesonide/formoterol as an anti-inflammatory reliever therapy [35].

In real-world practice, mild asthma patients are treated with an as-needed SABA reliever or with daily low-dose ICS maintenance therapy plus a SABA reliever. In the Novel START study, 668 patients with mild asthma were randomized to receive either as-needed albuterol 100 µg, two inhalations (SABA reliever as a continuation of the Step 1 treatment according to the 2017 GINA guidelines), budesonide 200 µg (ICS maintenance treatment) plus as-needed albuterol (Step 2 therapy of the GINA 2017 guidelines), or 200 µg/6 µg budesonide/formoterol as anti-inflammatory reliever therapy taken as-needed for a 52-week study period.

In this study, the rate of asthma exacerbations for budesonide/formoterol was lower compared with albuterol (51%) and similar to the twice-daily maintenance budesonide plus albuterol, despite a 52% reduction in the mean steroid dose with the single combination inhaler treatment [35]. In addition, severe exacerbation rate was lower with budesonide/formoterol as compared with as-needed albuterol and regular twice-daily budesonide. These data support the findings of the SYGMA 1 and 2 trials, highlighting the need for a critical re-examination of current clinical practice. Along with the results of the SYGMA trials, they provide convincing evidence of the advantages of the anti-inflammatory reliever strategy, particularly in real-life settings.

**Fig. 6:**
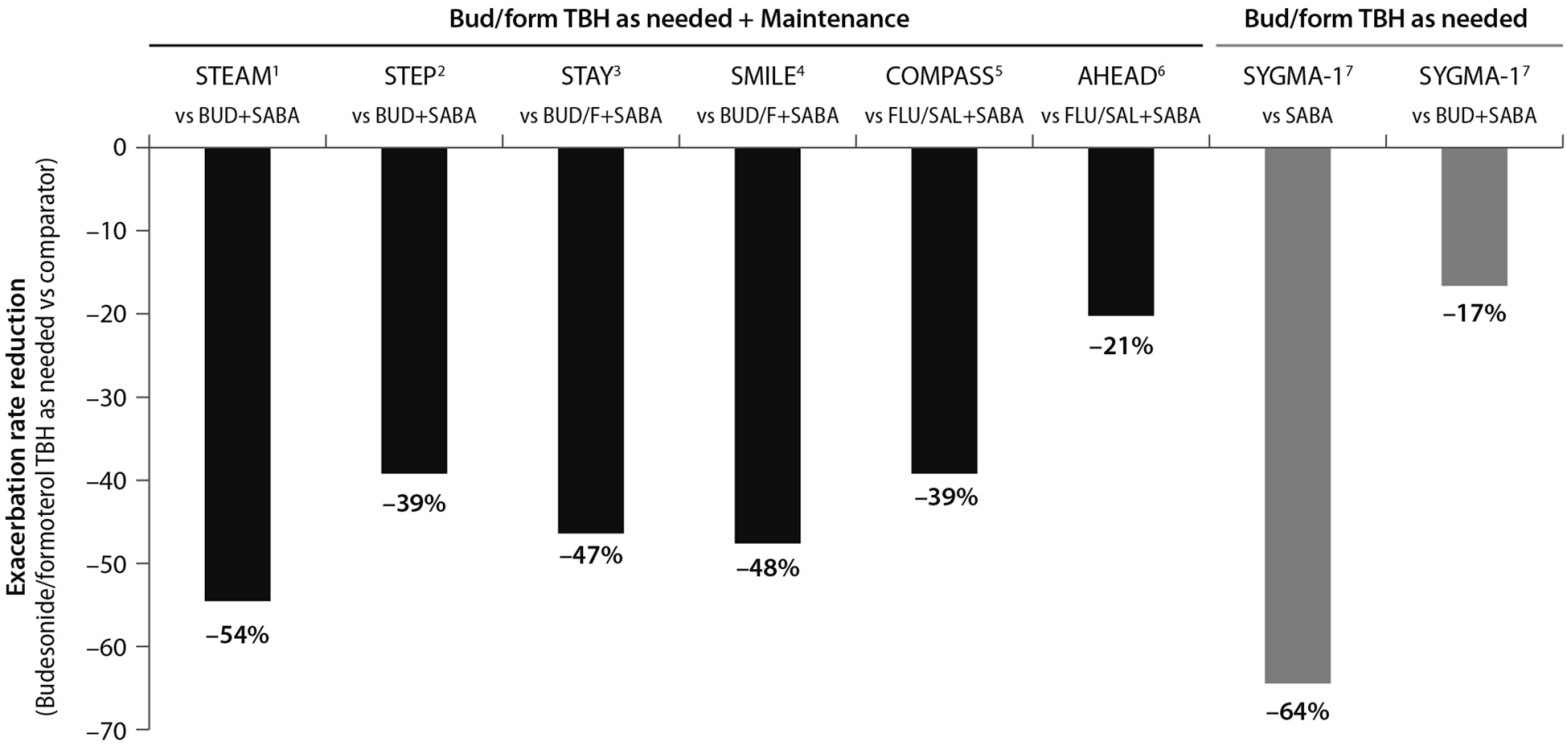
Risk reduction of severe asthma attack of anti-inflammatory reliever versus SABA across all levels of asthma severity. Bud = budesonide; form = formoterol; TBH = turbohaler. Data from: 1: [36]; 2: [37]; 3: [38]; 4: [28]; 5: [29]; 6: [30]; 7: [34] (Data source: [39])

The SYGMA 1, SYGMA 2 and the novel START studies complete the picture of the treatment strategies for asthma at any degree of severity, including mild asthma. A growing body of evidence shows that an anti-inflammatory reliever strategy, when compared with all other strategies with SABA reliever, consistently reduces the rate of exacerbations across all levels of asthma severity (Fig. 6) [28, 29, 34, 36–39].

**Fig. 7:**
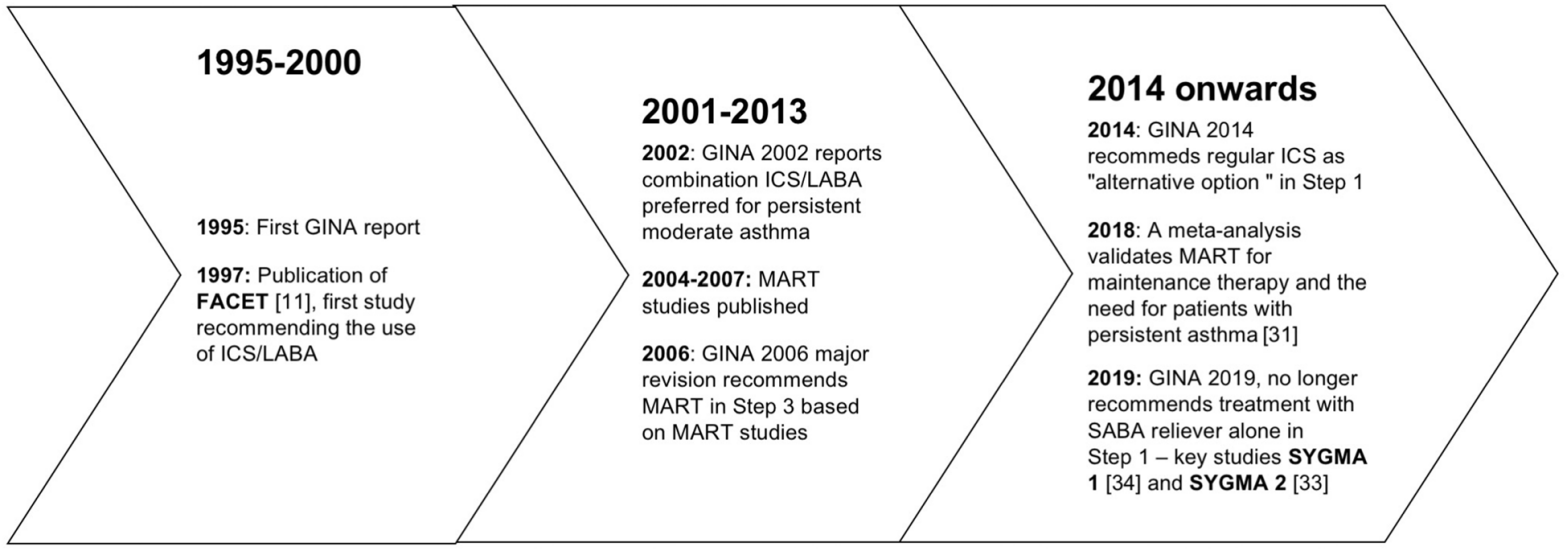
Timeline of key randomized controlled trials and meta-analyses providing the supporting evidence base leading to the Global Initiative for Asthma (GINA) 2019 guidelines. *GINA* global initiative for asthma, *MART* maintenance and reliever therapy, *SMART* single inhaler maintenance and reliever therapy

This evidence set the ground (Fig. 7) for the release of the 2019 GINA strategy updates. The document provides a consistent approach towards the management of the disease and aims to avoid the overreliance and overuse of SABAs, even in the early course of the disease. The 2019 GINA has introduced key changes in the treatment of mild asthma: for safety reasons, asthmatic adults and adolescents should receive ICS-containing controller treatment instead of the SABA-only treatment, which is no longer recommended.

In Step 1 of the stepwise approach to adjusting asthma treatment, the preferred controller option for patients with fewer than two symptoms/month and no exacerbation risk factors is low-dose ICS/formoterol as needed. This strategy is indirectly supported by the results of the SYGMA 1 study which evaluated the efficacy and safety of budesonide/formoterol as needed, compared with as-needed terbutaline and budesonide *bid* plus as-needed terbutaline (see above). In patients with mild asthma, the use of an ICS/LABA (budesonide/formoterol) combination as needed provided superior symptom control to as-needed SABA, resulting in a 64% lower rate of exacerbations (p = 0.07) with a lower steroid dose (17% of the budesonide maintenance dose) [34]. The changes extend to the other controller options as well. In the 2017 GINA guidelines, the preferred treatment was as-needed SABA with the option to consider adding a regular low-dose ICS to the reliever. In order to overcome the poor adherence with the ICS regimen, and with the aim to reduce the risk of severe exacerbations, the 2019 GINA document recommends taking low-dose ICS whenever SABA is taken, with the daily ICS option no longer listed.

Previous studies including the TREXA study in children and adolescents [40], the BASALT study [41] and research conducted by the BEST study group [32] have already added to the evidence that a low-dose ICS with a bronchodilator is an effective strategy for symptom control in patients with mild asthma. A recently published study in African-American children with mild asthma found that the use of as-needed ICS with SABA provides similar asthma control, exacerbation rates and lung function measures at 1 year, compared with daily ICS controller therapy [42], adding support to TREXA findings that in children with well controlled, mild asthma, ICS used as rescue medication with SABA may be an efficacious step-down strategy [40].

In Step 2 of the stepwise approach, there are now two preferred controller options: (a) a daily low-dose ICS plus an as-needed SABA; and (b) as-needed low-dose ICS/formoterol. Recommendation (a) is supported by a large body of evidence from randomized controlled trials and observations showing a substantial reduction of exacerbation, hospitalization, and death with regular low-dose ICS [7–9, 24, 43], whereas recommendation (b) stems from evidence on the reduction or non-inferiority for severe exacerbations when as-needed low-dose ICS/formoterol is compared with regular ICS [33, 34].

The new GINA document also suggests low-dose ICS is taken whenever SABA is taken, either as separate inhalers or in combination. This recommendation is supported by studies showing reduced exacerbation rates compared with taking a SABA only [32, 40], or similar rates compared with regular ICS [32, 40, 41]. Low-dose theophylline, suggested as an alternative controller in the 2017 GINA guidelines, is no longer recommended.

Airway inflammation is present in the majority of patients with asthma, and although patients with mild asthma may have only infrequent symptoms, they face ongoing chronic inflammation of the lower airways and risk acute exacerbations. The GINA 2019 strategy recognizes the importance of reducing the risk of asthma exacerbations, even in patients with mild asthma (Steps 1 and 2) [4]. In this regard, the new recommendations note that SABA alone for symptomatic treatment is non-protective against severe exacerbation and may actually increase exacerbation risk if used regularly or frequently [4].

The reluctance by patients to regularly use an ICS controller means they may instead try and manage their asthma symptoms by increasing their SABA reliever use. This can result in SABA overuse and increased prescribing, and increased risk of exacerbations.

As part of the global SABINA (SABA use IN Asthma) observational study programme, a UK study examined primary care records to describe the pattern of SABA and ICS use over a 10-year period in 373,256 patients with mild asthma [44]. Results showed that year-to-year SABA prescribing was more variable than that of ICS indicating that, in response to fluctuations in asthma symptom control, SABA use was increased in preference to ICS use. Furthermore, more than 33% of patients were prescribed SABA inhalers at a level equivalent to around ≥ 3 puffs per week which, according to GINA, suggests inadequate asthma control.

The problem of SABA overuse is further highlighted by two studies [45, 46], also as part of the SABINA programme. These analysed data from 365,324 patients in a Swedish cohort prescribed two medications for obstructive lung disease in any 12-month period (HERA).

The first study identified SABA overuse (defined as ≥ 3 SABA canisters a year) in 30% of patients, irrespective of their ICS use; 21% of patients were collecting 3–5 canisters annually, 7% were collecting 6–10, and 2% more than 11 [45]. Those patients who were overusing SABA had significantly more asthma exacerbations relative to those using < 3 canisters (20.0 versus 12.5 per 100 patient years; relative risk 1.60, 95% CI 1.57–1.63, p < 0.001). Moreover, patients overusing SABA and whose asthma was more severe (GINA Steps 3 and 4) had greater exacerbation risk compared with overusing patients whose asthma was milder (GINA Steps 1 and 2).

The second study found those patients using three or more SABA reliever canisters a year had an increased all-cause mortality risk relative to patients using fewer SABA canisters: hazard ratios after adjustment were 1.26 (95% CI 1.14–1.39) for 3–5 canisters annually, 1.67 (1.49–1.87) for 6–10 canisters, and 2.35 (2.02–2.72) for > 11 canisters, relative to patients collecting < 3 canisters annually [46].

The recently published PRACTICAL study lends further support to as-needed low-dose ICS/formoterol as an alternative option to daily low-dose ICS plus as-needed SABA, outlined in Step 2 of the guidelines [47]. In their one-year, open-label, multicentre, randomized, superiority trial in 890 patients with mild to moderate asthma, Hardy and colleagues found that the rate of severe exacerbations per patient per year (the primary outcome) was lower in patients who received as-needed budesonide/formoterol than in patients who received controller budesonide plus as-needed terbutaline (relative rate 0.69, 95% CI 0.48–1.00; p < 0.05). Indeed, they suggest that of these two treatment options, as-needed low-dose ICS/formoterol may be preferred over controller low-dose ICS plus as-needed SABA for the prevention of severe exacerbations in this patient population.

Step 3 recommendations have been left unchanged from 2017, whereas Step 4 treatment has changed from recommending medium/high-dose ICS/LABA [3] to medium-dose ICS/LABA; the high-dose recommendation has been escalated to Step 5. Patients who have asthma that remains uncontrolled after Step 4 treatment should be referred for phenotypic assessment with or without add-on therapy.

To summarise, the use of ICS medications is of paramount importance for optimal asthma control. The onset and increase of symptoms are indicative of a worsening inflammation leading to severe exacerbations, the risk of which is reduced by a maintenance plus as-needed ICS/LABA combination therapy. The inhaled ICS/bronchodilator combination is as effective as the regular use of inhaled steroids.

The efficacy of anti-inflammatory reliever therapy (budesonide/formoterol) versus current standard-of-care therapies in mild asthma (e.g. reliever therapy with a SABA as needed and regular maintenance controller therapy plus a SABA as-needed) has been evaluated in two randomized, phase III trials which confirmed that, with respect to as-needed SABA, the anti-inflammatory reliever as needed is superior in controlling asthma and reduces exacerbation rates, exposing the patients to a substantially lower glucocorticoid dose.

## Conclusions

A growing body of evidence shows that anti-inflammatory reliever strategy is more effective than other strategies with SABA reliever in controlling asthma and reducing exacerbations across all levels of asthma severity. A budesonide/formoterol therapy exposes asthma patients to a substantially lower glucocorticoid dose while cutting the need for adherence to scheduled therapy.

## Data Availability

Not applicable.

## Abbreviations

GINA: Global Initiative for Asthma
ICS: Inhaled corticosteroids
LABA: Long-acting beta-agonists
OCS: Oral corticosteroids
SABA: Short-acting beta-agonists
SMART: Single inhaler maintenance and reliever treatment
